# Retro-intraocular Lens Irrigation-aspiration to Prevent Capsular Block Syndrome at the Hands of Novice Surgeons

**DOI:** 10.7759/cureus.7285

**Published:** 2020-03-16

**Authors:** Harinder Singh Sethi, Mayuresh Naik, Abhinav Bhalla, Monika Kapur

**Affiliations:** 1 Ophthalmology, Vardhman Mahavir Medical College (VMMC) & Safdarjung Hospital, New Delhi, IND; 2 Ophthalmology, Hamdard Institute of Medical Sciences & Research (HIMSR) & Hakeem Abdul Hameed Centenary (HAHC) Hospital, New Delhi, IND; 3 Internal Medicine, Icahn School of Medicine at Mount Sinai, Mount Sinai Beth Israel, New York, USA

**Keywords:** retro-iol irrigation aspiration, posterior capsular opacification, capsular block syndrome

## Abstract

Retro-intraocular lens (IOL) irrigation-aspiration is of paramount importance in order to remove the viscoelastic substance from the retro-IOL space and to prevent any early post-operative capsular block syndrome. However, manoeuvring the IOL to reach the retro-IOL space may be difficult at the hands of novice surgeons despite the use of coaxial or bimanual irrigation-aspiration probes. We describe a simpler and safer technique in order to facilitate the removal of this retro-IOL viscoelastic substance using a 26-Gauge bent-cannula mounted on a 2-ml syringe. The fluid is injected forcefully along with sideways movement of cannula in a single-plane to displace the viscoelastic substance.

## Introduction

Cataract is the leading cause of preventable blindness in the world. Extra capsular cataract extraction is the norm wherein a significant part of the anterior lens capsule is removed leaving behind a rim of its remnant, which either forms the anterior extent of the resulting capsular bag or the posterior boundary of the sulcus. Phacoemulsification is the preferred extra capsular surgical technique for all types of lenticular opacities by most ophthalmic surgeons.

Capsular block syndrome (CBS) is a relatively uncommon complication of phacoemulsification with its incidence ranging between 0.3-1.6% [1-5]. It is characterized by hyperdistention of the capsular bag with accumulation of fluid in between the intraocular lens (IOL) and posterior capsule, as a result of the circumferential adherence between the remaining anterior capsule and IOL. It is classified on the basis of timing of occurrence by Miyake et al. into intra-operative, early, late post-operative CBS, and on the basis of the pathological process into non-cellular, inflammatory and fibrotic types by Kim and Shin in 2008 [6,7]. Early post-operative CBS is the more frequently encountered variety occurring after two weeks of surgery. Retro-IOL fluid accumulation with consequent anterior bowing of lens-iris diaphragm may result in secondary myopiasation, ocular hypertension or glaucoma depending upon the duration and severity of insult.

Retro-IOL irrigation-aspiration is of paramount importance in order to remove the viscoelastic substance from the retro-IOL space and to prevent any early post-operative capsular block syndrome. However, manoeuvring the IOL to reach the retro-IOL space may be difficult at the hands of novice surgeons despite the use of coaxial or bimanual irrigation-aspiration probes. We describe a simpler and safer technique in order to facilitate the removal of this retro-IOL viscoelastic substance using a 26-Gauge bent-cannula mounted on a 2-ml syringe.

## Materials and methods

Every cataract surgery was done using Infiniti® Vision System Ozil® phacoemulsification platform (Alcon, Fort Worth, TX). Balanced salt solution was used for the purpose of irrigation and hydro-dissection, while 2% Hydroxypropylmethyl-cellulose was the ophthalmic visco-surgical device (OVD) used as anterior chamber maintainer. A standard 2.8 mm clear corneal incision was made as main port in all the surgeries. A continuous curvilinear capsulorhexis of diameter 5.5 mm was done which was followed by a careful hydro-dissection. After the removal of lens nucleus, thorough cortical aspiration as well as polishing of the anterior capsular rim and posterior capsule was done using a coaxial irrigation-aspiration probe. The capsular bag was then distended with 2% Hydroxypropylmethyl-cellulose and a hydrophilic acrylic foldable IOL (Rayner C-flex® aspheric monofocal IOL with 5.75 mm optic) was injected into it.

After the IOL had completely opened inside the capsular bag, a 26-Gauge bent-cannula (available with HEALON GV® OVD, Abbott Medical Optics, Abbott Laboratories Inc. Abbott Park, Illinois, USA) mounted on a 2-ml syringe was introduced behind the IOL. The fluid is injected forcefully to displace the OVD along with sideways movement of cannula in a single-plane. The OVD that displaced from the retro-IOL space into the anterior chamber was then easily aspirated by the coaxial irrigation-aspiration probe. The wound was then closed with intra-stromal hydration and post-operative topical steroids on a tapered basis were continued for six weeks (Video 1).

**Video 1:** Operative video showing retro-IOL polishing and irrigation-aspiration IOL: Intraocular lens

## Results

Retro-IOL irrigation aspiration using 26-G bent canula was done in 50 cases of uncomplicated phacoemulsification by using a standard surgical protocol on the same phacoemulsification machine with implantation of the same posterior chamber IOL model by the same surgeon in the same settings.

Patients were followed up on first, third and seventh post-op days and thereafter at three weeks, two months and six months. Careful slit lamp examination to look for anterior chamber reaction, vitreous reaction, fluid accumulation behind the IOL and posterior capsular opacification (PCO) was done at every visit. Anterior segment OCT was done at one week, three weeks, two months and six months post-op. Early capsular block syndrome was diagnosed by post-op myopia, wherein a shallow anterior chamber and a distended capsular bag with IOL separation on anterior chamber optical coherence tomography (ASOCT) were found.

The capsulorrhexis was measured by a Castrovejo’s caliper to ensure a circular diameter of 5.5 mm as shown in the video. Cases in which the capsulorrhexis was irregular or greater than 5.75 mm were excluded from the study and final result analysis. Since the capsulorrhexis was created with a target diameter of 5.5 mm and the IOL has a 5.75-mm optic, it is deducible that that there would be some cases where there would be incomplete overlap which would allow egress of retained viscoelastic material. Such cases were excluded from the final results analysis so that only those cases with complete overlap of optic-rhexis margin were included in the study.

All 50 patients underwent uneventful phacoemulsification and reported no complications post-operatively. Of the two cases that were diagnosed to have early CBS, patient 1 had a post-op refraction of -2.5DS while patient 2 had a post-op refraction of -3DS. They underwent Nd-YAG laser capsulotomy at six weeks and then had an uneventful recovery. Patient 1 had uncorrected distant vision of 6/6 Snellen’s while patient 2 attained final best corrected visual acuity of 6/6 Snellen’s with -0.25DS.

## Discussion

Capsular block syndrome is a rare complication that may occur despite a neat continuous curvilinear capsulorrhexis (CCC) and an in-the-bag implantation of a posterior chamber IOL (PCIOL) but incomplete irrigation-aspiration of retro-IOL OVD [8]. It is primarily characterized by accumulation of fluid inside the capsular bag, specifically between the posterior capsule and IOL. Based on chronology, Miyake et al. proposed the first classification system for CBS in the year 1998 into intraoperative, early post-operative and late post-operative [6]. Intra-operative CBS occurs as a result of a rapid hydro-dissection, in hard cataracts and in high myopes, leading to the accumulation of fluid in the capsular bag and luxation of the crystalline lens. In severe cases, a posterior capsular rent may occur leading to the dislocation of the cataractous lens into the vitreous cavity. Late post-operative CBS is also known as lacteocrumenasia. It occurs 3-4 years after surgery and is identified by the deposition of white material behind the IOL inside the capsular bag, which has been shown to be rich in alpha crystallin protein [9,10].

Early post-operative capsular block syndrome can occur on the first post-operative day or in the first two weeks. Many mechanisms have been postulated to explain its occurrence. The likely pathogenic mechanism is the retention of ophthalmic viscosurgical device (OVD) in the retro-IOL space in the capsular bag that in turn creates an osmotic gradient across the capsule which works as a semi-permeable membrane [11]. Subsequently, the aqueous moves into the capsular bag. Since the volume of capsular bag is much larger than the IOL and undergoes no retraction, the IOL tends to be pushed forward by the fluid collecting behind it. Foldable lenses (acrylic/silicone) because of their structure get easily bowed anteriorly leading to their bio-adherence to the anterior capsule. According to Zacharias, capsulorhexes with adherences that compromise more than 70% of the border, act as a unidirectional valve allowing passage of liquid only into the capsular bag [12]. PMMA lenses on the other hand produce a stronger reaction upon their contact with anterior capsule leading to fibrosis and adhesion. Other factors which may be implicated in its pathogenesis are large optic IOL which makes aspiration of OVD difficult, small rhexis which accounts for its larger contact with anterior capsule, and use of OVDs like sodium hyaluronate and chondroitin sulphate which promote the osmotic ingress of aqueous. Retro-IOL irrigation ensures the complete removal/displacement of OVD from the retro-IOL space which is often inaccessible to direct irrigation-aspiration in the hands of novice surgeons. The displaced OVD can then be easily aspirated from the anterior chamber. Irrigation-aspiration using a co-axial or bimanual probe at the hands of a novice inexperienced surgeon may lead to posterior capsular tears (PCR) and vitreous incarceration into the anterior chamber along with their attending complications/sequelae. In our technique, during the procedure the surgeon continuously keeps injecting fluid and keeps the cannula in touch with the posterior surface of the IOL. This helps to avoid posterior capsular damage.

The implications of the technique are multifold. Reduction in incidence of early post-operative CBS results in decrease in incidence of early post-operative myopia and thereby prevent erroneous diagnoses like IOL surprise. The burden on the medical system in order to diagnose and work-up erratic myopic post-operative refractive errors will decrease and improve patient satisfaction markedly. Early-CBS would require to be treated by Nd-YAG laser capsulotomy while Nd-YAG laser capsulotomy has its own complications, the most important among them being macular edema. Thus, a meticulous surgical approach of retro-IOL irrigation with a simple 26-G bent canula may help reduce the incidence of both CBS, at the hands of novice inexperienced surgeons, especially in developing countries such as India, where not only can most patients not afford expensive IOLs (multipiece IOL in case of an inadvertent PCR) but also the availability of Nd-YAG laser machines may appear to be a far-fetched prospect.

## Conclusions

Capsular block syndrome is a rare complication that may occur following a continuous curvilinear capsulorrhexis (CCC) and in-the-bag implantation of a posterior chamber IOL. It is primarily characterized by accumulation of fluid inside the capsular bag, specifically between the posterior capsule and IOL. Retro-IOL irrigation ensures the complete removal/displacement of OVD from the retro-IOL space which is often inaccessible to direct irrigation-aspiration. The displaced OVD can then be easily aspirated from the anterior chamber. Apart from this, the retro-IOL irrigation with sideways movement of cannula aids in cleaning the posterior surface of the IOL off any residual surface debris which get attached to the posterior surface of the IOL during insertion within the cartridge.

## References

[1] Vélez M, Velásquez LF, Rojas S, Montoya L, Zuluaga K, Balparda K (2014). Capsular block syndrome: a case report and literature review. Clin Ophthalmol.

[2] Khattak A (2017). Bilateral early capsular block syndrome following implantation of the new trifocal toric lens. Oman J Ophthalmol.

[3] Koh JS, Song YB, Wee WR, Han YK (2016). Recurrent late-onset fibrotic capsular block syndrome after neodymium-yttrium-aluminum-garnet laser anterior capsulotomy: a case report. BMC Ophthalmol.

[4] Kim HD, Kim JM, Jung JJ (2017). Complete occlusion of anterior capsulorhexis after uneventful cataract surgery, treated with YAG laser capsulotomy. BMC Ophthalmol.

[5] Grover DS, Goldberg RA, Ayres B, Fantes F (2012). Treatment of late-onset capsular distension syndrome with a neodymium:YAG laser peripheral iridotomy and anterior capsulotomy. J Cataract Refract Surg.

[6] Miyake K, Ota I, Ichihashi S, Miyake S, Tanaka Y, Terasaki H (1998). New classification of capsular block syndrome. J Cataract Refract Surg.

[7] Kim HK, Shin JP (2008). Capsular block syndrome after cataract surgery: clinical analysis and classification. J Cataract Refract Surg.

[8] Ho JD, Lee JS, Chen HC, Ho CL, Chen SN (2003). Early postoperative capsular block syndrome. Chang Gung Med J.

[9] Kucukevcilioglu M, Hurmeric V, Erdurman FC, Ceylan OM (2011). Imaging late capsular block syndrome: ultrasound biomicroscopy versus Scheimpflug camera. J Cataract Refract Surg.

[10] Eifrig DE (1997). Capsulorhexis-related lacteocrumenasia. J Cataract Refract Surg.

[11] Sugiura T, Miyauchi S, Eguchi S (2000). Analysis of liquid accumulated in the distended capsular bag in early postoperative capsular block syndrome. J Cataract Refract Surg.

[12] Zacharias J (2000). Early postoperative capsular block syndrome related to saccadic-eye-movement-induced fluid flow into the capsular bag. J Cataract Refract Surg.

